# Comprehensive Phylogenetic Analysis of Bacterial Group II Intron-Encoded ORFs Lacking the DNA Endonuclease Domain Reveals New Varieties

**DOI:** 10.1371/journal.pone.0055102

**Published:** 2013-01-23

**Authors:** Nicolás Toro, Francisco Martínez-Abarca

**Affiliations:** Grupo de Ecología Genética, Estación Experimental del Zaidín, Consejo Superior de Investigaciones Científicas (CSIC), Granada, Spain; Centro de Biología Molecular Severo Ochoa (CSIC-UAM), Spain

## Abstract

Group II introns are self-splicing RNAs that act as mobile retroelements in the organelles of plants, fungi and protists. They are also widely distributed in bacteria, and are generally assumed to be the ancestors of nuclear spliceosomal introns. Most bacterial group II introns have a multifunctional intron-encoded protein (IEP) ORF within the ribozyme domain IV (DIV). This ORF encodes an N-terminal reverse transcriptase (RT) domain, followed by a putative RNA-binding domain with RNA splicing or maturase activity and, in some cases, a C-terminal DNA-binding (D) region followed by a DNA endonuclease (En) domain. In this study, we focused on bacterial group II intron ORF phylogenetic classes containing only reverse transcriptase/maturase open reading frames, with no recognizable D/En region (classes A, C, D, E, F and unclassified introns). On the basis of phylogenetic analyses of the maturase domain and its C-terminal extension, which appears to be a signature characteristic of ORF phylogenetic class, with support from the phylogeny inferred from the RT domain, we have revised the proposed new class F, defining new intron ORF varieties. Our results increase knowledge of the lineage of group II introns encoding proteins lacking the En-domain.

## Introduction

Group II introns are self-splicing RNAs that act as mobile retroelements and are thought to be the ancestors of nuclear spliceosomal introns [1]–[4]. Group II introns were initially identified in the mitochondrial and chloroplast genomes of lower eukaryotes and plants, but were subsequently found in bacteria and archaea [5]–[9]. A typical group II intron consists of a highly structured RNA that folds into a conserved three-dimensional structure consisting of six distinct double-helical domains, DI to DVI [10]. Most bacterial group II introns have a multifunctional intron-encoded protein (IEP) ORF within DIV [8]. Group II IEPs have an N-terminal reverse transcriptase (RT) domain homologous to retroviral RT sequences, followed by a putative RNA-binding domain with RNA splicing or maturase activity (domain X) and, in some cases, a C-terminal DNA-binding (D) region followed by a DNA endonuclease (En) domain [11], [12]. Three main classes (IIA, IIB and IIC) of group II introns have been described on the basis of conserved ribozyme structures, with additional subclasses displaying specific structural variations (i.e., IIA1, IIA2, IIB1 and IIB2) [8], [13]. It is generally accepted that group II intron RNAs have coevolved with their IEPs, and phylogenetic analyses of group II intron-encoded ORFs has resulted in their classification into several groups (A, B, C, D, E, F, CL1 [chloroplast-like 1], CL2 [chloroplast-like 2] and ML [mitochondrion-like]). Nomenclatures of intron subclasses based on ribozyme structure or the IEP are widely used, but these classifications overlap to some extent. This has led to proposals for a single classification based on the coevolution of the two intron components, RNA and IEP (i.e. intron class D, with a IIB-like RNA structure, is known as IIB3 in this system) [7]. According to the IEP-based phylogenetic nomenclature, bacteria contain introns from all phylogenetic classes, whereas organelles contain only introns of the CL and ML classes. Class A, C, D, E and F introns encode proteins with no En domain. It is generally thought that the En domain of group II introns was acquired by an RT/maturase IEP [9], [14], and it has recently been suggested that this domain may have been acquired only once, by a common ancestor of B, E, ML and CL introns, subsequently being lost from class E [15].

Class F is the most recently identified bacterial ORF phylogenetic class. It currently includes a small group of bacterial group II introns with a typical class IIB RNA secondary structure, similar in overall organization to the introns of class E [16]. However, phylogenetic analysis (RT and X domains) of class F ORFs has shown relatively low levels of bootstrap support (<70%), increasing to 73% only when the unclassified introns basal to this group are excluded [16]. Simon *et al*. [15] performed a large-scale phylogenetic analysis of group II introns with sequence data for both the conserved RNA structure and the intron-encoded reverse transcriptase. They found that each ORF class, including class F, was a robust clade, but that the relationship between classes remained ambiguous. Furthermore, amino-acid sequence-based phylogenies were found to be sensitive to taxon sampling, with similar sets of introns often producing different tree topologies with different levels of support for the conflicting nodes. In the most recent compilation of group II introns by Zimmerlýs group [17], which was based on 397 bacterial ORF-encoding introns (sequences >95% identical are given the same name and listed only once within a given species) from the database for bacterial group II introns (http://webapps2.ucalgary.ca/~groupii/index.html#), class F contained only seven introns, whereas 10 unclassified introns also lacked a recognizable D/En region.

We previously showed that the C-terminal portion of class D RT/maturase proteins constitutes a distinctive, characteristic signature of the ORF class, which has been conserved throughout evolution. It constitutes a group-specific functionally important protein region contributing to both maturase function and intron mobility [18]. Here, on the basis of consensus maturase and C-terminal amino-acid sequences, with additional support from the phylogeny inferred from the larger RT-domain, we extend current knowledge of intron ORF subclasses with no recognizable D/En region. Our results call into question the proposed new class F, but reveal the existence of additional group II intron ORF varieties.

## Materials and Methods

### Search for additional class D and new types of RT/maturase ORFs lacking the endonuclease domain

We searched non redundant (GenBank, RefSeq, EMBL, DDBJ and PDB) databases for class D group II IEPs, using BlastP or TBlastN, with the consensus amino-acid sequence obtained from a multiple sequence alignment (MSA) of domain X and the C-terminal extension of 35 bacterial class D ORFs [18]. Additional class D ORF sequences were identified and the final dataset for phylogenetic analysis included 67 that were ≤75% identical (Table S1). We also searched for new types of RT/maturase ORFs lacking the endonuclease domain, using the amino-acid consensus sequence obtained from the MSA of domain X and the C-terminal extension for reported new class F and unclassified introns downloaded from the database for bacterial group II introns. The search was carried out as for the identification of class D ORFs, but with BlastP only. The additional sequences identified in the first search were then included in the alignment to obtain a refined consensus sequence, which was used to identify more distant relatives. All the previously reported class F and unclassified introns were identified among the Blast hits, together with 27 new, related ORF sequences. The final dataset for phylogenetic analysis included 50 ORF sequences that were ≤75% identical (Table S2). All these analyses were carried out with Geneious Pro software (Biomatters Ltd.).

### Phylogenetic analyses

We focused on group II intron ORF phylogenetic classes containing only reverse transcriptase (RT)/maturase open reading frames (ORFs), with no recognizable D/En region (classes A, C, D, E, F and unclassified introns), using all available sequences from the database until 07-03-2012, and the new sequences identified in this work. The phylogenetic relationships between unclassified and class F intron ORFs available from the database were first investigated by comparing phylogenetic trees estimated by maximum likelihood methods from alignments of sequences for the RT and for the X-domain plus its C-terminal extension. These alignments were generated with Geneious Pro software (Biomatters Ltd.) and Clustal W.

The alignment used for the phylogenetic analysis of intron ORFs lacking a recognizable D/En region was initially based on the X-domain and C-terminal sequences. It covered a mean of 130 amino acids corresponding to gap-free lengths of 174 unique sequences that were ≤75% identical (Figure S1). The only exceptions were the class A ORF sequences E.c.I4 and S.f.I1 (99% identity), and the Ni.haI1 (class F) and Ni.haI2 (unclassified) ORFs (99% identity). The larger RT-domain, containing a mean of 257 amino acids, was also used for the phylogenetic analysis of a subset of 123 unique sequences with recognizable RT amino-acid sequences (Figure S2). We included representatives [15] of subclasses B, CL and ML in other analyses. An unrooted tree and 100 boostraps were generated with PhyML [19], [20], implemented in Geneious Pro (Biomatters Ltd.), with the WAG amino-acid substitution model. Note that different substitution models (i.e. WAG and LG) delivered trees with the same topology. The GUIDANCE method [21] was used to determine alignment confidence scores and to remove aligned positions considered unreliable according to a given cutoff. GUIDANCE is freely available for use from http://guidance.tau.ac.il. The species delimitation plugin for Genious [22] was used to calculate the inter-species divergence and Rosenberǵs reciprocal monophyly P(AB), a test for taxonomic distinctiveness, according to the null hypothesis that monophyly is a chance outcome under a model of random coalescence in a single group [23].

Other types of analysis were performed to investigate the influence of the various phylogenetic estimation methods on our results. We carried out Bayesian analysis with the parallel version of MrBayes 3.1 [24] implemented in Geneious Pro (Biomatters Ltd.). Two independent runs of four chains were completed for 1,100,000 Metropolis-coupled Markov chain Monte Carlo (MCMCMC) generations, using the default priors for model parameters, the WAG model as the rate matrix (fixed) and the gamma model for between-site rate variation. Trees were sampled every 200 generations, and 110,000 samples were discarded as the “burn-in”, to produce a 50% majority-rule consensus tree.

### Identification of group II intron boundaries

Nucleotide sequences (4–5 kb) containing putative intron-encoded ORFs were analyzed with Mfold for the detection of potential group II intron RNA structures [25]. We searched for intron boundaries with the bioinformatics tool from the intron database [17]. Most of the ORFs presented intron boundaries that were identified with this tool. However, some of the candidate introns of the new varieties described here had no predicted intron boundaries. In these cases, a conserved and, presumably, catalytic domain V was identified, generally just downstream from the ORF sequence. Clustal W alignment of the ORF surrounding the RNA sequence (generally 2.0–2.5 kb in size), together with an examination of putative exon junctions, made it possible to define the boundaries and a consensus group II intron structure (not shown) for these new varieties.

## Results and Discussion

### Relationships between class F and unclassified group II intron-encoded ORFs

**Figure 1:**
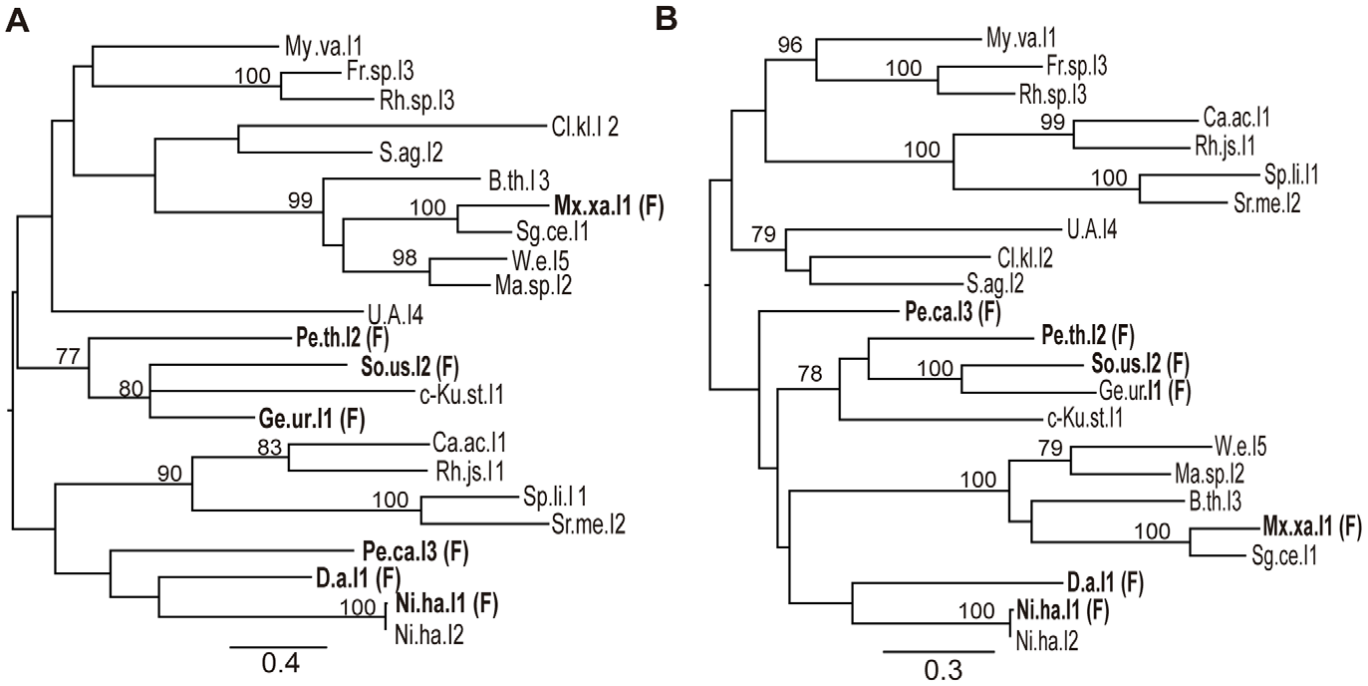
ORF phylogeny based on alignments of the amino-acid sequences of unclassified and class F intron-encoded ORFs. (*A*) Phylogenetic tree based on the X-domain and its C-terminal extension. (*B*) Phylogenetic tree based on the RT-domain. MLE consensus trees based on MSAs generated with Clustal W, using the WAG substitution model. Trees are rooted at their midpoint. Bootstrap results (≥75%) are given at each node (PhyML) and the rate of amino-acid substitutions per site is shown at the bottom of each tree. Introns assigned to class F are indicated and highlighted in bold typeface.

As mentioned above, Simon *et al*. [16] reported a phylogenetic analysis based on the RT/maturase sequence, in which unclassified introns were basal to class F, decreasing statistical support for this clade. We investigated the possible relationships between these intron ORFs, by carrying out a phylogenetic analysis of all the class F and unclassified intron ORFs reported in the database, by the maximum likelihood estimation method [19], [20]. The phylogenetic trees (Fig. 1) show that the class F intron M.xa.I1 lies within a well supported group that also contains the unclassified introns Sg.ce.I1, W.e.I5, Ma.sp.I2 and B.th.I3, with bootstrap values of 99% (X-domain and its C-terminal extension, Fig. 1A) and 100% (RT-domain, Fig. 1B). In addition, the related class F introns So.us.I2 and Ge.ur.I1 belong to a cluster that includes another class F intron (P.th.I2) and the unclassified intron c-Ku.st.I1. Moreover, the class F introns D.a.I1 and Ni.ha.I1 cluster with the unclassified intron Ni.ha.I2. The position of the class F intron Pe.ca.I3 is uncertain in both trees. The phylogenetic trees thus indicate a lack of statistical support for the grouping together of class F introns.

### Updated compilation of bacterial group II intron-encoded ORFs lacking the endonuclease domain

Bacterial group II intron ORF phylogenetic classes containing only reverse transcriptase/maturase open reading frames, with no recognizable D/En region, belong to class A, which had only three almost identical (99% identity) members, class C, class E and class D. Moreover, class E was split into two clades, E1 and E2 [16], whereas other ORFs in the intron database were classified simply as “class E”. We therefore carried out a comprehensive phylogenetic analysis of intron ORFs and investigated the phylogeny of the proposed class F and the other unclassified introns further, by generating a larger dataset of related ORFs, as described in the materials and methods. In this analysis, we identified 27 new ORFs (Table S2) not reported in the intron database, that were related to the proposed class F and unclassified introns; only 14 of these ORFs had a complete RT-X sequence. The 50 ORFs included in the final dataset originated from 37 different bacterial species and six subdivisions of Eubacteria (Acidobacteria, 1; Actinobacteria, 16; Bacteroidetes, 1; Firmicutes, 10; Proteobacteria, 20 and Verrumicrobia, 1). One ORF was found in a nonculturable environmental archaea sample.

We also updated the compilation of class D intron-encoded ORFs and identified 41 not reported in the intron database, 18 of which had a complete RT-X sequence (Table S1). The 67 class D ORFs included in the final dataset originated from 49 different bacterial species belonging to seven subdivisions of Eubacteria (Acidobacteria, 1; Actinobacteria, 7; Bacteroidetes, 3; Chlorobi, 4; Firmicutes, 9; Proteobacteria, 33 and Thermotogae, 1); four originated from nonculturable environmental bacteria, one from an archaea (Euryarchaeota), one from a nonculturable environmental archaea sample and, surprisingly, one was found in a draft plant genome sequence (*Ricinus communis*).

We investigated whether the newly identified intron ORFs had an associated ribozyme, by analyzing the secondary structure motifs typical of group II introns. Determinations of the secondary structure of domains I, II and III upstream from the ORFs and of the characteristic downstream domains, V and VI (Table S1 and Table S2), revealed an associated group II intron RNA structure. Moreover, 13 of the 27 newly identified ORFs related to former class F or unclassified introns were full-length, as were 14 of the 41 new class D ORFs.

### Identification of new types of intron-encoded ORF based on the maturase domain and its C-terminal extension

Phylogenetic analyses were performed on an amino-acid sequence alignment (Figure S1) for domain X and its C-terminal extension for 174 bacterial group II introns, by the maximum likelihood estimation method. The initial set of intron ORFs included all class A, C, D, E, F and unclassified introns reported in the database and the new introns identified in this work. The final dataset used for the analyses contained only selected sequences that were ≤75% identical (with a few exceptions, as indicated).

**Figure 2:**
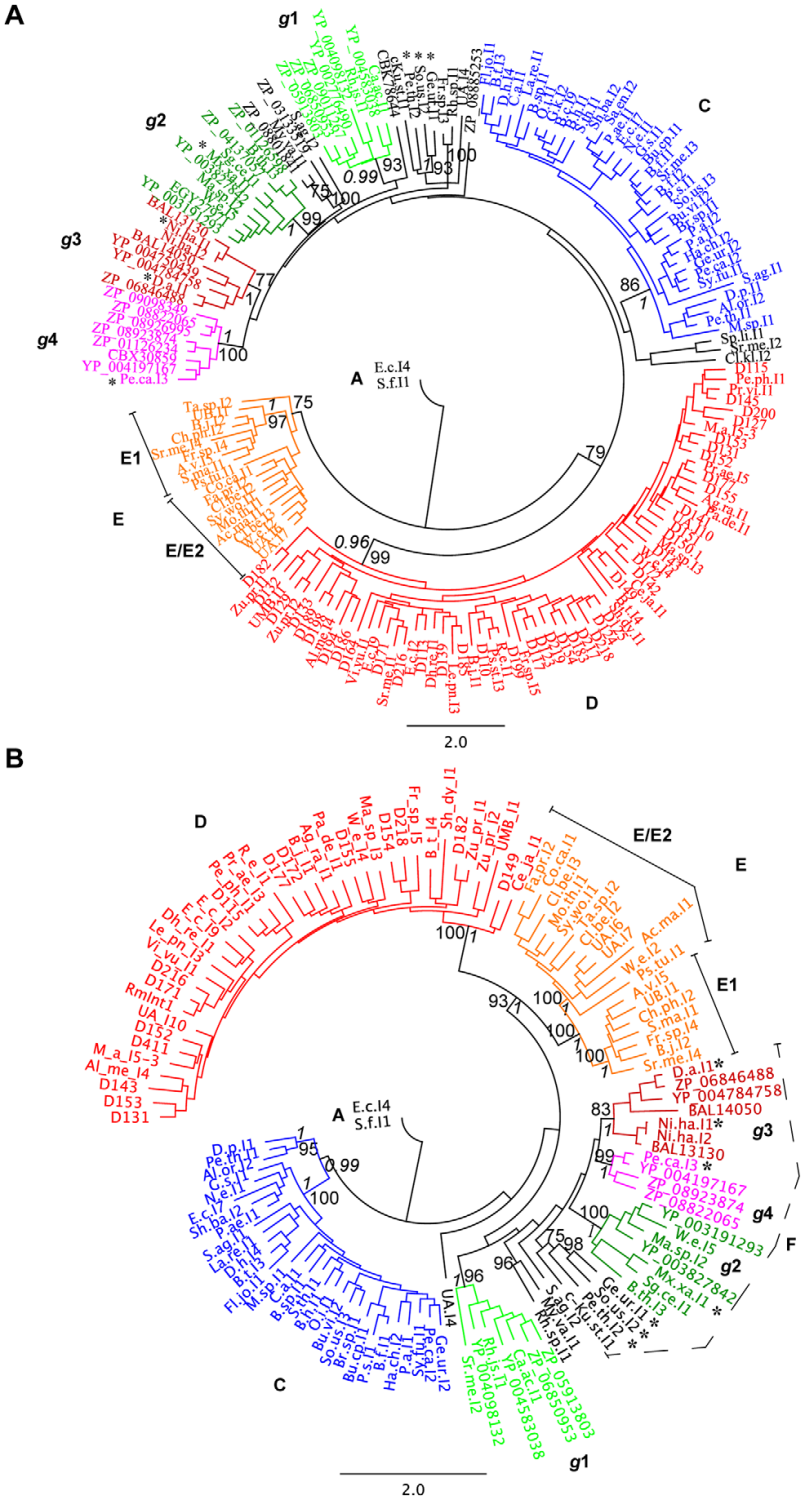
Phylogeny of group II intron ORF classes lacking a recognizable D/En region. (*A*) Phylogenetic tree based on the alignment of the X-domain and its C-terminal extension. (*B*) Phylogenetic tree based on the alignment of the RT-domain (0–7). Consensus unrooted trees estimated by ML methods are presented as a radial phylogram. The phylogenetic ORF classes are indicated and the rate of amino-acid substitutions per site is shown at the bottom of each tree. The branches of the major classes are also color-coded to improve visualization of the major clades. Bootstrap results (≥75%) are given for each node (PhyML). Posterior probability values (≥0.96) for Bayesian analysis of the major phylogenetic ORF classes are also indicated in italics. Introns previously classified as phylogenetic ORF class F are indicated by an asterisk.

The estimated phylogenetic tree supports the existence of the previously described classes C, D and E (Fig. 2A), but not that of class F. As previously reported by Simon *et al*. [16], we found that class E could potentially be split into two clades, E1 and E/E2, but statistical support was strong only for E1, with a 97% bootstrap value and a high posterior probability (1). Ta.sp.I2 appeared to be the most divergent ORF within class E.

Class D, C and other ORFs branched from a common node with a high bootstrap value (79%), but a low posterior probability (0.51). The phylogenetic tree for the domain X and C-terminal amino-acid sequences also suggested the existence of additional varieties of introns, with strong statistical support (bootstrap value >75%) and a high posterior probability (≥0.99). One of these new groups (hereafter referred to as *g*1), supported by a bootstrap value of 93%, contains eight different ORFs, including those of the Ca.ac.I1 and Rh.js.I1 introns previously described as “unclassified”. A second group (hereafter referred to as *g*2) with strong support (99% boostrap) contained 10 different ORFs, including those of the Mx.xa.I1 intron, which had previously been assigned to class F, and the unclassified introns W.e.I5, Ma.sp.I2, Sg.ce.I1 and B.th.I3. A third group (hereafter referred to as *g*3), with a high posterior probability (1) and high bootstrap values (77%), contained eight introns, including the class F introns D.a.I1 and Ni.ha.I1 and the previously unclassified intron Ni.ha.I2. Finally, a fourth group (hereafter referred to as *g*4) with strong statistical support (100% boostrap) had eight members, including the class F intron Pe.ca.I3. The relationships between these groups and the major classes remain unclear, because the deeper branches of the tree have yet to be resolved. Notably, maximum likelihood estimation on the basis of a reduced multiple sequence alignment (MSA; 84 informative positions encompassing only the conserved X-domain without the C-terminal extension), with the removal of unreliable columns by the GUIDANCE [21] method (cutoff 0.427 and a guidance alignment score of 0.827309) provided strong statistical support for *g*4 (96%) and *g*2 (94%). Likewise, high levels of statistical support were obtained for class D (73%) and class E (82%), whereas the short alignment decreased the bootstrap value for classes C and *g*1 to 57% and 65%, respectively, and provided no significant support for *g*3 (not shown). In the short MSA tree, phylogenetic resolution was lower, but nonetheless consistent with the monophyly of *g*2 and *g*4.

### Comparison of maturase and C-terminal phylogeny with that based on the RT-domain

**Figure 3:**
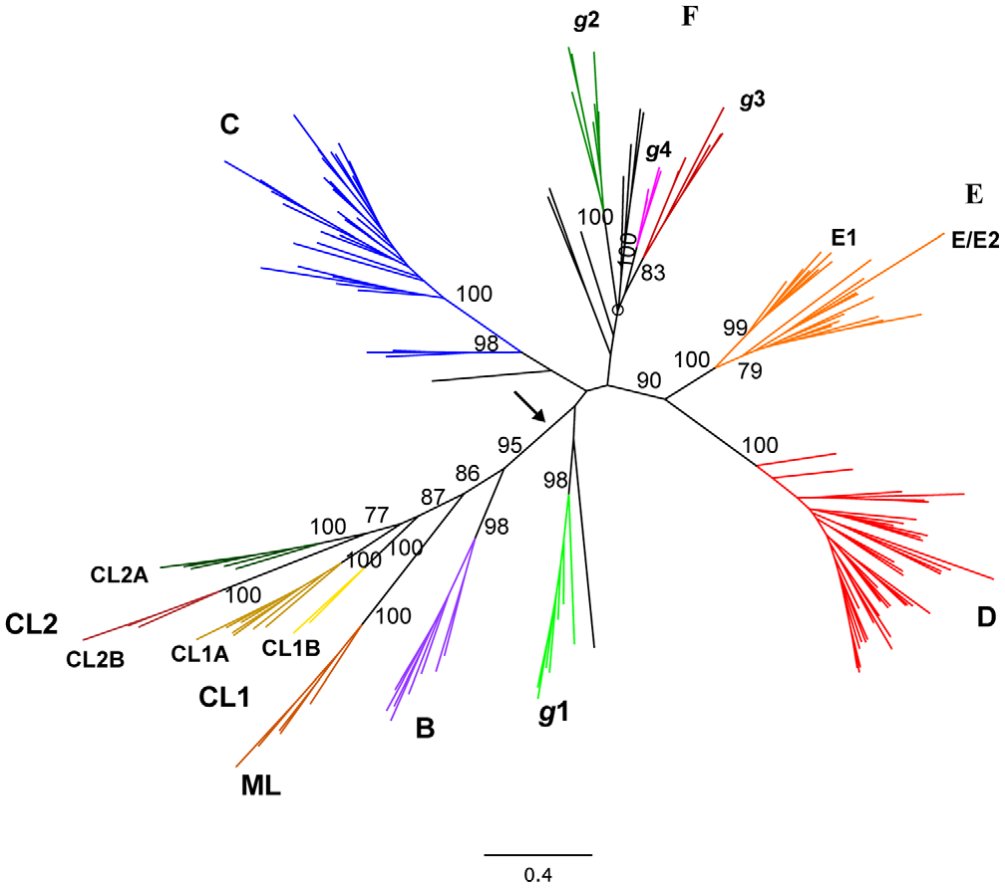
RT domain-based phylogeny of group II intron ORFs. To the MSA alignment used for Fig. 2B, representatives of classes B: En.fmI3, B.a.I2, En.fm.I1, E.f.I3, B.me.I1, C.d.I1, G.k.I1, and B.c.I5; CL1A: A.v.I1, P.p.I2, E.c.I5, Th.e.I1, B.t.I1, Sh.sp.I1, Ms.b.I1; CL1B: B.me.I2, and B.a.I1; CL2A: Tr.e.I2, G.v.I1, Ns.p.I4, Cs.p.I1, and C.w.I1; CL2B: C.w.I6, Ns.p.I2, and Ns.p.I1; and ML: Ba.fr.I1, B.t.I2, N.a.I2, and L.l.I1 used by Simon *et al*. [15] were added (RT 0–7). The corresponding accession numbers can be obtained from the bacterial group II intron database. A consensus unrooted tree is shown and major ORF classes and the new groups are labeled. Bootstrap results (≥75%) are given for each node (PhyML). The arrow indicates the common node of the lineage of group II introns with an En domain in their IEPs. The circle indicates the possible node grouping together class F introns.

The conserved RT-domain has a larger number of readily alignable characters than the X-domain and its C-terminal extension. It therefore contains more informative positions for phylogenetic analyses. We investigated whether the phylogeny inferred from this domain was consistent with the definition of the new varieties of intron-ORFs described above. Phylogenetic analysis was performed on a subset of 123 unique sequences with a recognizable RT amino-acid sequence, based on the corresponding MSA (Figure S2), by the maximum likelihood estimation method and Bayesian inference. In general, the inferred phylogeny was congruent with that obtained with the maturase and C-terminal amino-acid sequences. The estimated phylogenetic tree (Fig. 2B) supported the existence of the previously described classes C, D and E, with 100% bootstrap values and high posterior probabilities (1), but not that of class F. Strong statistical support was also obtained for the new groups *g*1-4 (posterior probability of 1, boostrap value >80%). Nevertheless, this phylogeny identifies several specific features. Some of the class C intron ORFs (D.p.I1, Al.or.I2 and Pe.th.I1) lie outside the main node, suggesting that they might be divergent ORFs within this class. Intron Sr.me.I2, which remained unclassified in the maturase tree (note that its ORF lacks the C-terminal extension), clustered within *g*1 in the RT tree. The intron ORFs clustered in *g*2 contained a characteristic FADD consensus motif, rather than YADD, in the RT5 domain. There was strong support for sister clade status for classes D and E (posterior probability of 1, bootstrap value of 93%). This phylogeny was not altered by the addition to the MSA of intron ORF RT-sequences representative of classes B, CL1, CL2 and ML, all of which have an En-domain in their IEPs. This intron lineage branched from a strongly supported (95% bootstrap) node, suggesting that the En domain was acquired only once, in a common ancestor (Fig. 3).

The interspecies distance between the currently recognized classes C, D and E (Fig. 2B) ranged from 2.591 (D:E) to 3.333% (D:C) divergence, and all had significant P(AB) values (P<10^−5^). The interspecies distance of the monophyletic *g*1-4 ranged from 1.325 (*g*3:*g*4) to 2.740% (*g*1:*g*2) divergence, but only *g*1 and *g*2 have significant P(AB) values, with *g*1 identified as the most divergent clade.

## Conclusions

We have increased the number of known introns in bacteria and added to knowledge concerning the lineage of group II introns encoding RT/maturase proteins lacking a D/En region. We have identified several new intron ORF varieties and revised the definition of the previously proposed class F. We found that the introns used to established class F [15], [16] belonged to different monophyletic groups, and that only group F members Pe.thI2, So.us.I2 and Ge.ur.I1 appeared to form a distinct clade. Thus, the proposed class F should be reconsidered. The new intron varieties identified here could be classified into four different well supported monophyletic groups (*g*1-4). Group *g*1, which contains no introns previously classified as class F, displayed a significant interspecies distance and may constitute a separate ORF-based phylogenetic subclass. By contrast, *g*2-4 each included group F members and shared a common node with a basal group containing the rest of the former class F introns (Ge.ur.I1, So.us.I2 and Pe.th.I2), and the unclassified intron c-Ku.st.I1. The grouping together of *g*2-4 and the small basal F group does not seem to be supported by high bootstrap values in the tree. However, we nevertheless propose that class F should be represented by the four former clades until their relationships can be resolved.

The relationships between the intron ORF classes of the intron lineage lacking the En-domain remain unclear, because the deeper branches of the tree have yet to be resolved. Nevertheless, the phylogeny based on the RT-domain suggests that classes E and D may be sister clades. We therefore obtained no evidence to support the hypothesis that class E introns form part of the lineage of introns with an En domain in their IEPs [15]. Instead, our data suggest that the En domain was acquired only once, by the common ancestor of classes B, CL and ML.

The large number of bacterial genome sequences already available and the continual production of additional sequence data provide the basis for successful analyses of group II intron diversity. We expect even greater intron diversity in bacteria to be detected through future surveys of sequence databases, providing insight into the importance of these catalytic RNAs and retroelements in bacterial evolution.

## Supporting Information

Figure S1 **MSA based on the maturase and C-terminal extension sequences.**
(TIF)

Figure S2 **MSA based on the RT-domain (RT0-7).**
(TIF)

Table S1 Class D group II introns.
(XLS)

Table S2 New varieties of group II introns lacking the En domain.
(XLS)
