# Supplementary figures and images for: Comprehensive Phylogenetic Analysis of Bacterial Group II Intron-Encoded ORFs Lacking the DNA Endonuclease Domain Reveals New Varieties
Source: PLoS One. 2013 Jan 23;8(1):e55102. doi: 10.1371/journal.pone.0055102 (PMC3552965; doi:10.1371/journal.pone.0055102)

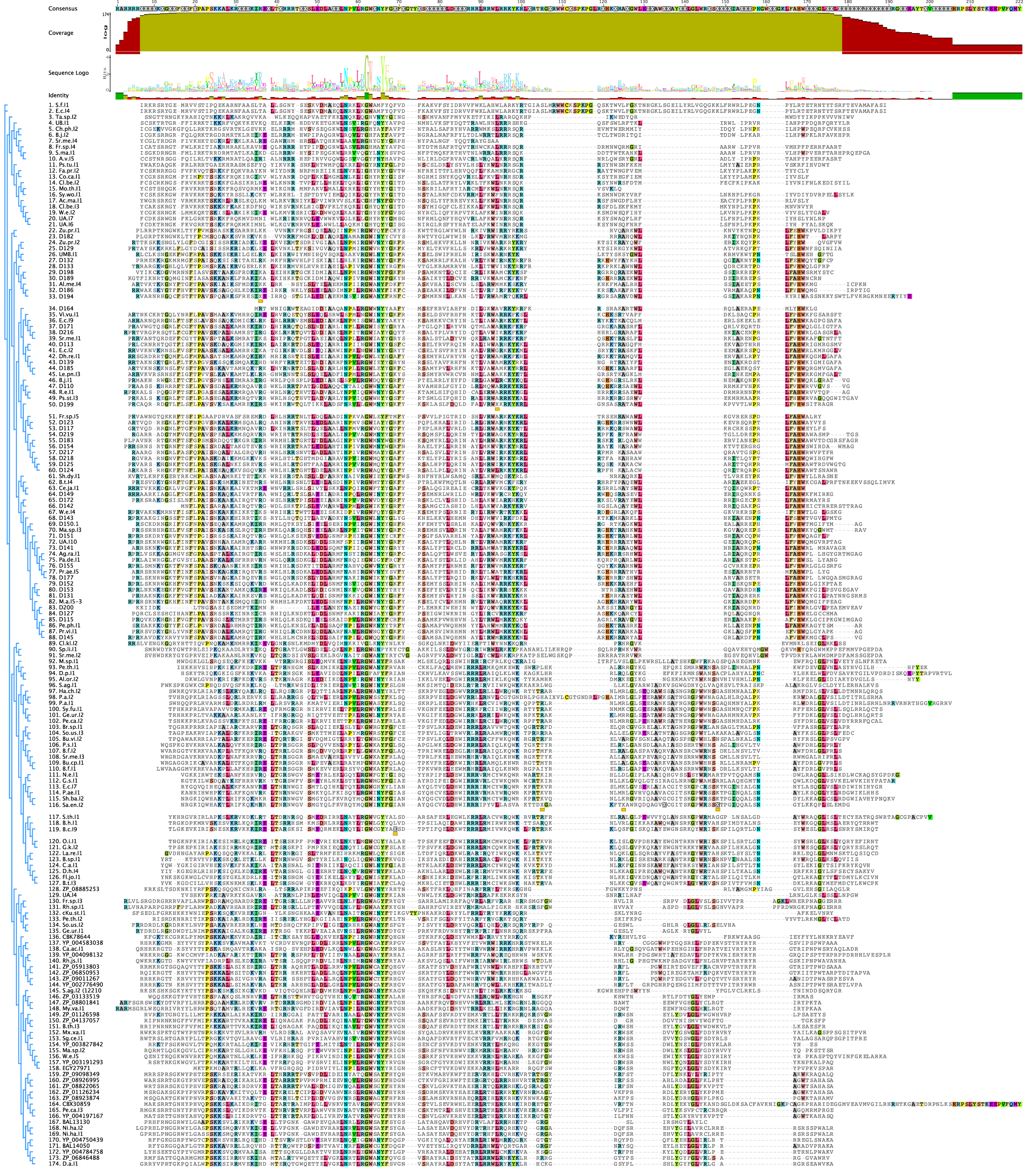

Supplement: Figure S1 — MSA based on the maturase and C-terminal extension sequences. (TIF) [file pone.0055102.s001.tif]

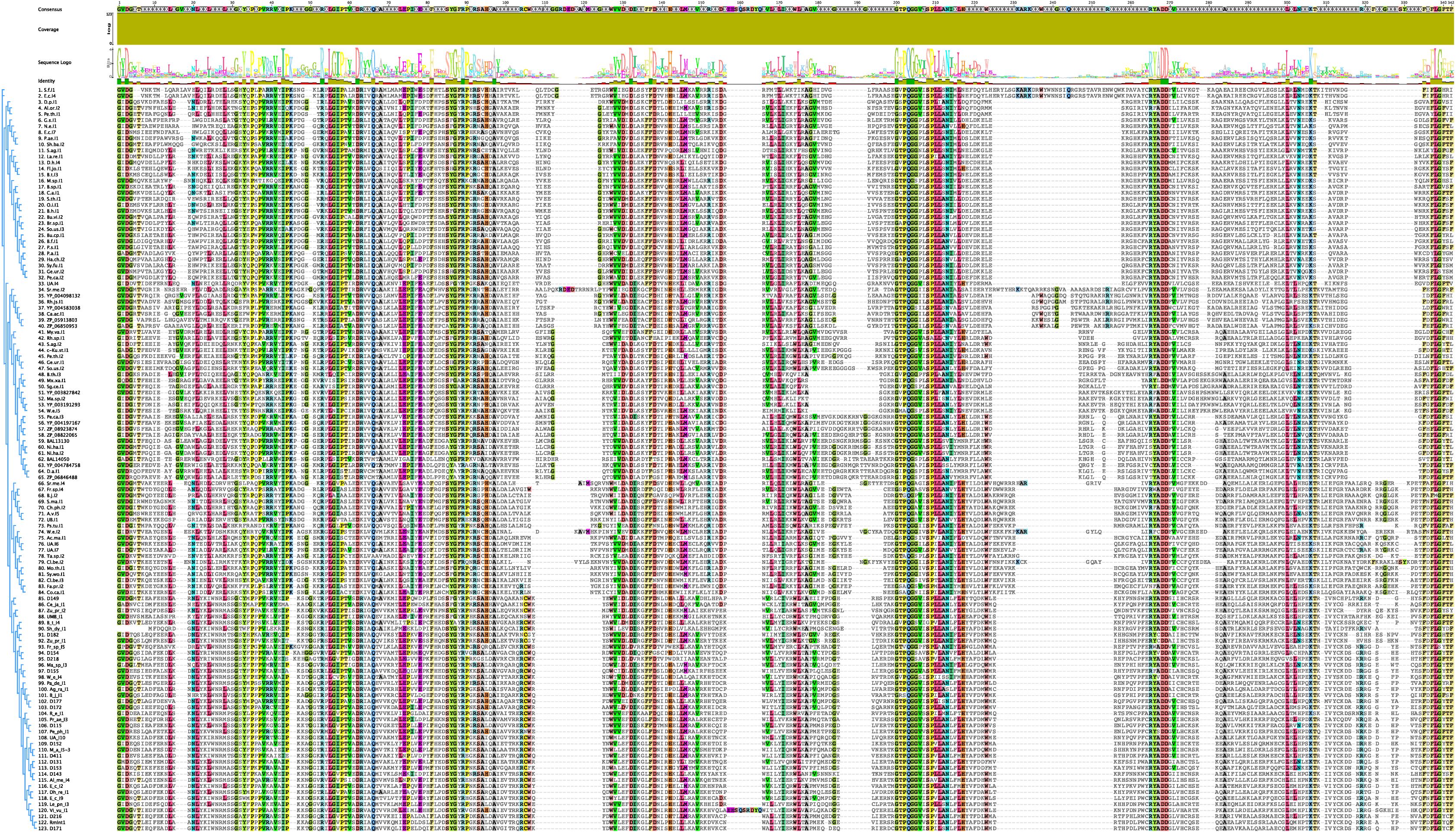

Supplement: Figure S2 — MSA based on the RT-domain (RT0-7). (TIF) [file pone.0055102.s002.tif]
